# Therapeutic Administration of the Chemokine CXCL1/KC Abrogates Autoimmune Inflammatory Heart Disease

**DOI:** 10.1371/journal.pone.0089647

**Published:** 2014-02-27

**Authors:** Kurt Bachmaier, Sophie Toya, Asrar B. Malik

**Affiliations:** Department of Pharmacology, College of Medicine, University of Illinois at Chicago, Center for Lung and Vascular Biology, Chicago, Illinois, United States of America; Centre d'Immunologie de Marseille-Luminy, CNRS-Inserm, France

## Abstract

Myocarditis, often due to an aberrant immune response to infection, is a major cause of dilated cardiomyopathy. Microbial pattern recognition receptors, such as TLRs, orchestrate the cytokine and chemokine responses that augment or limit the severity of myocarditis. Using the mouse model of experimental autoimmune myocarditis (EAM), in which disease is induced by immunization with a heart-specific self peptide and the agonist to multiple TLRs, complete Freund's adjuvant, we found that increased serum concentrations of the chemokine CXCL1/KC correlated directly with decreased severity of myocarditis. To directly test whether CXCL1/KC caused the amelioration of myocarditis, we treated mice, after challenge with heart-specific self peptide, with exogenous recombinant CXCL1/KC. We found that the administration of recombinant mouse CXCL1/KC completely abrogated heart inflammatory infiltration and cardiomyocyte damage. Moreover, we show that TLR4 signaling is required to increase serum protein concentrations of CXCL1/KC in EAM, and we demonstrate that the administration of the TLR4 agonist LPS significantly decreased severity and prevalence of EAM and reduced the number of heart-specific self peptide reactive effector T cells. These findings reveal a novel function of CXCL1/KC in the context of organ-specific autoimmune disease that may prove useful for the treatment of inflammatory conditions that underlie human heart disease.

## Introduction

In myocarditis, infectious agents instigate cellular and humoral immune reactions that lead to myocardial inflammation and cardiomyocyte damage [1]–[3]._ENREF_2_ENREF_3 Myocardial inflammation can result in acute and chronic dilated cardiomyopathy (DCM) [2], [4]. The clinical picture of myocarditis varies from asymptomatic to cardiogenic shock [2]. Current therapy for myocarditis is supportive, aimed at management of clinical symptoms of both myocarditis and cardiomyopathy [3]. At the time of diagnosis, it is difficult to assert definitively an aberrant immune reaction to infection or a precise pathogenetic mechanism of myocarditis. The routine use of immunosuppressive agents in patients with myocarditis and cardiomyopathy is not indicated since it is not clear in which cases immunosuppression is likely to be beneficial [2]. Although there is a high rate of spontaneous improvement in acute myocarditis and cardiomyopathy, patients who develop chronic DCM have an abysmal 5-year survival rate of below 50% [5].

In the present study, we used a well established murine model [6] to gain fresh insights into the pathogenesis of myocarditis that can be exploited for novel therapeutic strategies. Experimental autoimmune myocarditis (EAM) is a T_H_17 mediated disease whose induction depends on dendritic cells (DCs) that encounter autoantigen and present it in an immunogenic fashion in conjunction with MHC II molecules [7]. In *in vitro* models, TLRs, and specifically TLR4, have a crucial role in this process [8]. Histopathologically, EAM is characterized by an inflammatory infiltrate that consists mostly of lymphocytes and macrophages [6]. Heart macrophages are thought to exert both protective and detrimental effects in the course of EAM [9], [10]. The environmental clues that determine the function of effector T cells and inflammatory macrophages in EAM remain poorly understood. In particular, the role of the molecular pattern recognition receptor TLR4 in induction, maintenance and resolution of EAM is controversial [11]–[13].

Therefore, in the present study, we examined the effect of TLR4 signaling on prevalence and severity of EAM. We found that TLR4 signaling significantly ameliorated histopathological disease severity, accelerated its resolution, and that TLR4 is required for the induction of CXCL1/KC expression. Based on this endogenous induction of circulatory CXCL1/KC in the course of EAM, we hypothesized that treatment with exogenous recombinant CXCL1/KC would abrogate inflammation in EAM.

## Results

**Figure 1:**
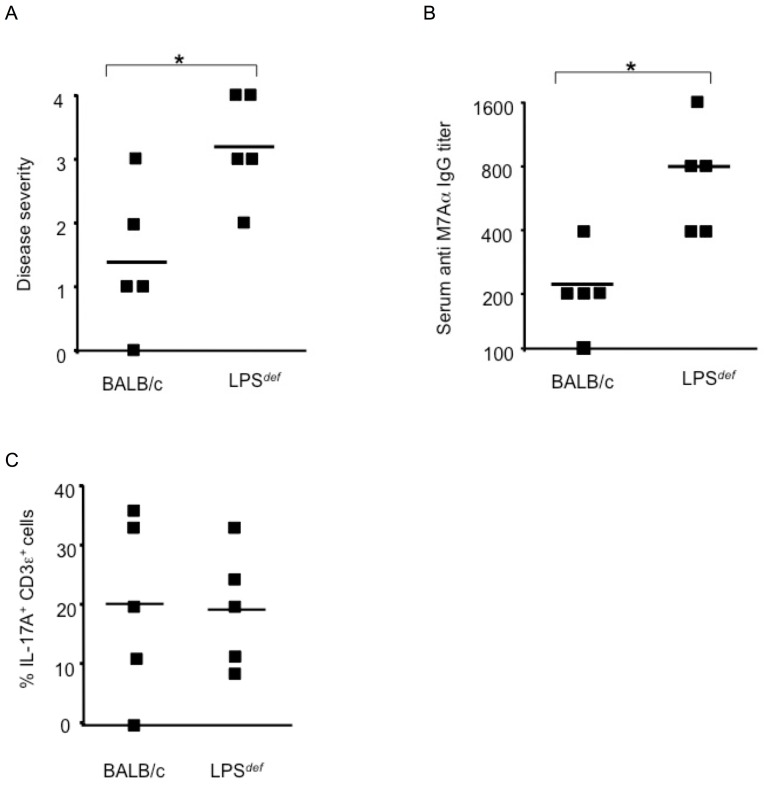
*LPS* ^def^ mice are highly susceptible to induction of autoimmune inflammatory heart disease. Genetic loss of TLR4 function leads to severe autoimmune myocarditis in mice challenged with heart-specific autoantigen and complete Freund's adjuvant (CFA), a complex mixture of TLR agonists. (A) *LPS* ^def.^ mice lacking functional TLR4 developed significantly more severe autoimmune myocarditis that wild type BALB/c control mice. Histopathological disease severity, as described in Methods, was determined 21 days after the initial immunization with heart-specific M7Aα peptide and CFA. * p<0.05. One representative result out of 5 independent experiments is shown. (B) Serum IgG autoantibodies reactive to heart specific epitope M7Aα were determined 21 days after initial immunization with heart-specific M7Aα peptide and CFA. * p<0.05. One representative result out of 5 independent experiments is shown. (C) Heart inflammatory infiltrate. CD3ε^+^ T cells expressing IL-17A were evaluated 21 days after initial immunization with heart-specific M7Aα peptide and CFA. Squares represent the percentage of IL-17A^+^ cells per CD3ε^+^ T cells as determined by immunohistochemistry in heart-sections from individual mice, lines indicate mean values. * p<0.05. One representative result out of 5 independent experiments is shown. (D) Representative photomicrograph of heart section from a *LPS* ^def^ mouse immunized with autoantigen and CFA. Inflammatory infiltrate consisting mostly of mononuclear cells is present throughout the myocardium often surrounding necrotic cardiomyocytes (arrow). Original magnifications x10 and x200 are shown. Hearts were analyzed 21 days after initial immunization with heart-specific M7Aα peptide in CFA. Staining was with hematoxylin and eosin (H&E). (E) Histopatholgy in a heart section from a BALB/c mouse immunized with heart-specific M7Aα peptide in CFA. Inflammatory infiltrate consisting mostly of mononuclear cells is present as an inflammatory focus (arrow). Original magnifications x100 is shown. (F) *LPS* ^def.^ mice fail to resolve autoimmune myocarditis by day 28 after the initial autoantigen challenge but not wild type BALB/c control mice. Histopathological disease severity, as described in Methods, was determined 28 days after the initial immunization with heart-specific M7Aα peptide and CFA. * p<0.05. Squares represent individual mice, lines indicate mean values. One representative result out of 3 independent experiments is shown. Student's t Test was used for statistical analysis.

In EAM, the T helper type 17 (T_H_17) mediated activation and expansion of CD4^+^ T cells reactive to heart epitopes is dependent on the TLR adaptor protein MyD88 in antigen presenting cells (APCs) [9], [14]–[16], _ENREF_81demonstrating the role of TLRs in the pathogenesis of the disease. Hence we used mice carrying a mutation in the gene for the LPS receptor, TLR4, that renders the mice resistant to LPS (*LPS* ^def^) but highly susceptible to Gram-negative infection [17] to address the role of TLR4 and LPS in EAM. We injected the cardiac-specific M7Aα peptide emulsified in complete Freund's adjuvant (CFA), a complex mixture of mycobacterial TLR ligands [18], [19], into mice, housed under specific pathogen free conditions, to induce the disease. We found that *LPS* ^def^ mice challenged with M7Aα in CFA developed significantly more severe myocarditis than wild type BALB/c control mice 21 days after the initial immunization (Figure 1A) and *LPS* ^def^ mice had significantly higher titers of auto-antibodies reactive to the M7Aα epitope than controls (Figure 1B). The histopatholgy of EAM in *LPS* ^def^ mice was characterized by monuclear cell infiltrate, consisting mostly of CD68^+^ macrophages and IL17A^+^ T cells, and cardiomyocyte damage (Figure 1C,D) similar to EAM in BALB/c control mice (Table 1, Figure 1E). EAM in wild type mice is largely resolved by day 28 after the initial immunization with heart specific antigen in CFA [20]. Hearts of *LPS* ^def^ mice immunized with M7Aα peptide in CFA, however, had active inflammatory foci even by day 28 after the initial immnization whereas hearts of wild type control mice were free of inflammatory cells and showed no histopathological signs of cardiomyocyte damage (Figure 1F). These findings indicate that the presence of functional TLR4 ameliorated the severity of disease and accelerated the resolution of myocarditis.

**Table 1:** CXCL1/KC prevents the induction of iNOS protein expression in hearts of mice challenged with cardiac-specific α-myosin-heavy chain-derived peptide M7Aα.

Immunization	Treatment	iNOS^+^Cardiomyocytes	CD68^+^	CD68^+^iNOS^+^
None	Saline	1.2±1.0	n.d.	n.d.
M7Aα	Saline	45.5±12.1*	59.3±9.6	54.8±12.5
M7Aα	CXCL1/KC	2.5±2.0	n.d.	n.d.

Frequency of positive cells in heart sections from mice immunized with M7Aα in CFA 21 days after the initial immunization. Percentage of cells (±SD) staining for iNOS in cardiomyocytes, or CD68 and iNOS in inflammatory cells, was calculated after counting cells per visual field at magnification of x160 on sections from hearts of 3 mice per group. A minimum 100 cells in at east 10 different visual fields was evaluated per heart. One result representative of 3 independent experiments is shown. * p<0.05 when compared to the other groups. n.d., cells were not detected in sufficient numbers, i.e., there were <100 cells per visual field.

**Figure 2:**
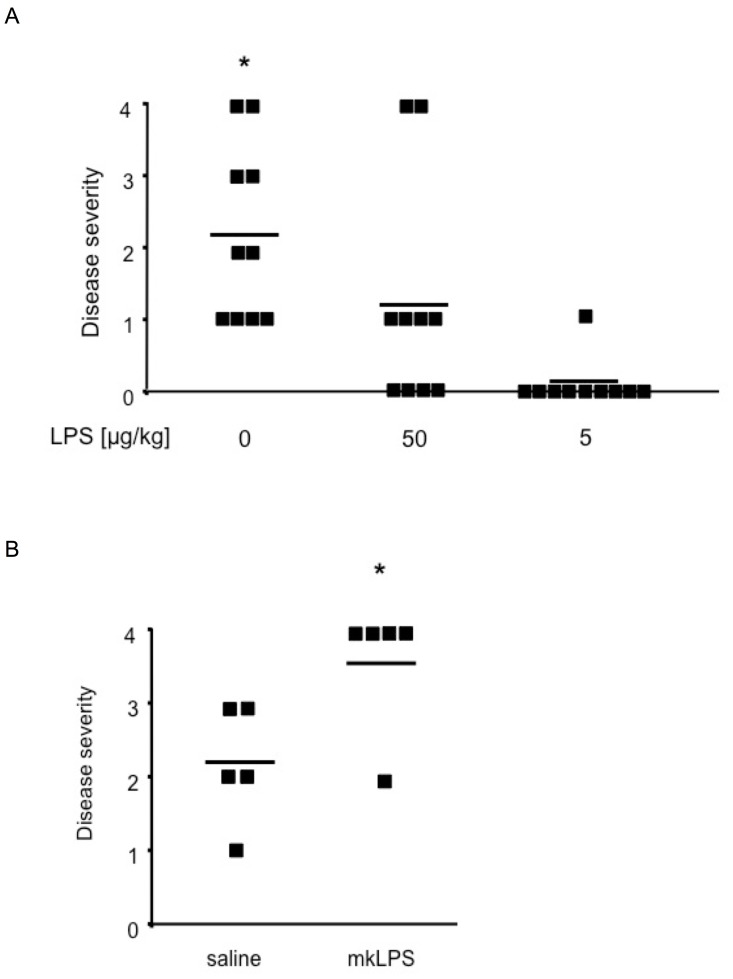
TLR4 signaling is crucial for prevalence and severity of experimental autoimmune myocarditis. (A) Systemic administration of low dose LPS curbed the induction of experimental autoimmune heart disease. Wild type BALB/c mice treated with low doses LPS developed myocarditis with significantly reduced severity and prevalence compared to saline treated controls. Mice were immunized with M7Aα peptide in CFA and treated by intraperitoneal injection 3 times, on days 5, 9, and 13 after the initial immunization, with the indicated doses of highly pure LPS or saline. Squares represent individual mice, lines indicate mean values. * p<0.05 when compared to other groups using ANOVA for multiple-sample comparisons (Bonferroni). (B) Antagonizing the LPS receptor TLR4 significantly increased severity of autoimmune myocarditis. mkLPS, which lacks the myristoyl fatty acid moiety of lipid A, antagonized TLR4 signaling through direct interaction with TLR4. Mice immunized with M7Aα peptide in CFA and mkLPS developed significantly more severe myocarditis than control mice receiving M7Aα peptide in CFA and saline. Disease severity was determined by histopathology 21 days after the initial immunization. Squares represent individual mice, lines indicate mean values. * p<0.05 by Student;s t-test. One representative result out of 4 independent experiments is shown.

Heart M7Aα peptide and CFA TLR ligands are internalized and processed through the DC endophagosome after subcutaneous injection of the emulsion, i.e., antigen and TLR ligands are localized in the same cellular compartment, which is required for effective antigen presentation [8]. To test whether the systemic availability of the TLR4 ligand LPS during the induction phase of the disease effected prevalence and severity of EAM, we injected wild type BALB/c mice intraperitoneally with LPS after the initial challenge with M7Aα peptide in CFA, at doses that induced no signs of inflammation or leukocyte activation when given on their own (data not shown). Mice immunized with M7Aα peptide in CFA and treated with 3 doses of LPS (50 µg/kg) showed significantly reduced disease prevalence and severity when compared to saline treated control mice (Figure 2A). Mice treated with 5 µg/kg LPS showed a further reduction in disease severity and prevalence (Figure 2A). LPS given at doses of 500 µg/kg, while significantly reducing TLR4 cell surface expression on circulating leukocytes, had no effect on EAM severity and prevalence when compared to saline treated control mice (data not shown).

Since *LPS* ^def^ mice may have altered T cell receptor (TCR) repertoire and compromised immune cell function [17] which might influence susceptibility to EAM, we evaluated the absence of TLR4 signaling by an alternative method; i.e., using a pharmacological inhibitor of TLR4, mkLPS [21]. mkLPS prevents, through direct interaction with TLR4, activation of downstream signaling pathways by TLR4 agonists [22]. We found that BALB/c wild type mice immunized with M7Aα peptide in CFA developed significantly more severe EAM when TLR4 signaling was inhibited with mkLPS than control mice immunized with M7Aα peptide in CFA in the absence of mkLPS (Figure 2B). These pharmacological and genetic data clearly show that ablation of TLR4 signaling aggravated EAM and that intraperitoneal LPS treatment ameliorated EAM in mice.

**Figure 3:**
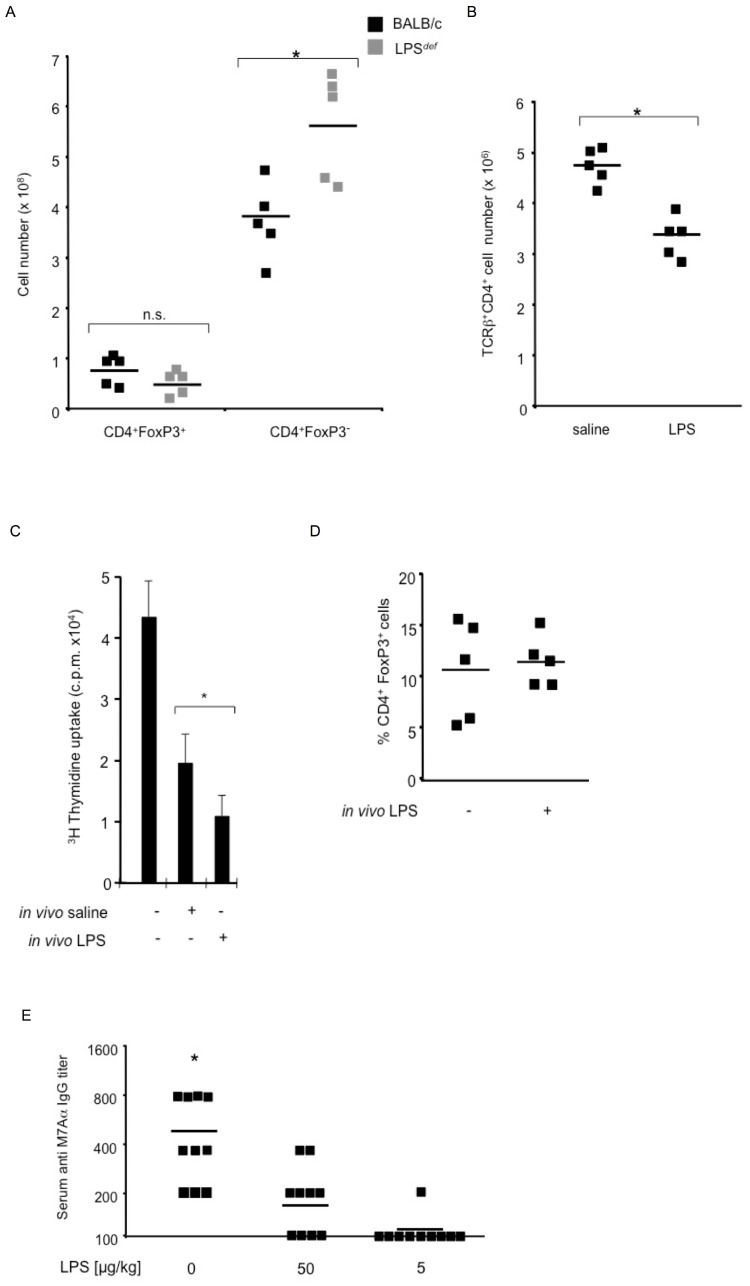
LPS-induced TLR4 signaling inhibits T and B effector lymphocyte function in experimental autoimmune heart disease. (A) *LPS* ^def^ mice have similar numbers of TCRβ^+^CD4^+^Foxp3^+^ T_regs_ after challenge with M7Aα peptide in CFA. The number of effector CD4^+^Foxp3^−^ T_effs_ was significantly increased in spleens of *LPS* ^def^ mice after autoantigen challenge. Squares represent individual mice, lines indicate mean values. (B) LPS treatment *in vivo* dampened the proliferation of heart Ag-specific effector T cells *ex vivo*. An equal number of T_effs_, isolated from spleens, either of mice immunization with M7Aα in CFA and treated either with three injections of either LPS (5 µg/kg) or from saline treated controls, was cultured with γ-irradiated syngeneic stimulator splenocytes pulsed with the M7Aα peptide. Squares represent the number of cultured cells obtained from individual mice, lines indicate mean values. (C) Suppression of proliferation of T cells (5x10^4^) from naïve mice stimulated with anti-CD3 ε (1 µg/ml) plus anti-CD28 (0.1 µg/ml) antibodies in the presence of pre-cultered T cells (1x10^5^, derived from mice immunized with M7Aα in CFA and treated either with three injections of either LPS (5 µg/kg) or saline. * p<0.05 by Student;s t-test. A representative result from 3 experiments is shown. (D) Systemic administration of low doses of LPS significantly reduced the concentration of IgG reactive to heart-specific epitope M7Aα. Mice were immunized and treated as described in Figure 2. * p<0.05 when compared to other groups using ANOVA for multiple-sample comparisons (Bonferroni. One representative result out of 4 independent experiments is shown.

In a mouse model, depleting CD4^+^CD25^+^Foxp3^+^ regulatory T cells (T_regs_) leads to severe multi-organ inflammation, and, similar to EAM, to autoimmune myocarditis with high-titers of anti-myosin autoantibodies [23]. LPS can directly activate T_reg_ function [24], and T_reg_ function can be blocked, indirectly involving IL-6, by the activation of TLRs on APCs [25]. To address the mechanism by which TLR4 signaling ameliorated EAM, we examined T_reg_ cells in *LPS* ^def^ mice. We found that the number of TLR4 expressing TCRβ^+^ CD4^+^Foxp3^+^ T lymphocytes was comparable between *LPS* ^def^ mice. After challenge with M7Aα peptide in CFA, the number of T_regs_ present in the spleen remained comparable between genotypes (Figure 3A). The number of effector CD4^+^Foxp3^−^ T lymphocytes (T_effs_) was, however, significantly increased in spleens of *LPS* ^def^ mice after autoantigen challenge (Figure 3A). These data suggested that TLR4 signaling limits the expansion of effector CD4^+^Foxp3^−^ T lymphocytes.

We therefore analyzed the effects of LPS treatment *in vivo* on the proliferation of heart Ag-specific effector T cells *ex vivo*. An equal number of T_effs_, isolated either from spleens of mice immunization with M7Aα in CFA and treated either with three injections of LPS (5 µg/kg) or saline, was cultured with γ-irradiated syngeneic stimulator splenocytes pulsed with the M7Aα peptide. We found that *in vivo* LPS treatment significantly reduced the *ex vivo* proliferation of T_effs_ reactive to the heart-specific M7Aα peptide (Figure 3B). To determine the cause of reduced proliferation of T_effs_ reactive to the heart-specific M7Aα peptide we performed suppression assays with T cells obtained from LPS-treated and saline-treated mice immunized with M7Aα peptide in CFA. We cultured T cells from LPS-treated or saline-treated mice, immunized with M7Aα peptide in CFA, as described above, with γ-irradiated syngeneic stimulator splenocytes pulsed with the M7Aα peptide. We added 1×10^5^ cultured T cells to T cells (5×10^4^) from naïve syngeneic donors, to U-bottom 96 well plates with anti-CD3ε and anti-CD28 for 48 h. T cells from LPS-treated donors inhibited the proliferation of naïve T cells significantly more efficient that T cells from saline treated controls (Figure 3C). Simultaneously, at the start of the suppressor assay, we determined the phenotype of the cultured T cells by determinig the percentage of FoxP3^+^ cells among CD4^+^ T cells. We found that T cells from LPS-treated donors and from saline treated controls gad similar numbers of FoxP3^+^ cells (Figure 3D). These data suggests that *in vivo* treatment with LPS leads to the generation of FoxP3^+^ T_reg_ cells that are more effective in inhibiting T_eff_ proliferation than in controls. Together, these data demonstrate that the mechanism of TLR4 amelioration of EAM involves the expansion of T_effs_ and, accordingly the ratio of T_regs_ to T_effs_ was significantly increased in LPS treated mice.

Since autoantibody production to heart epitopes is dependent on T_eff_ function [26] we next measured the effect of LPS treatment on the production of IgG reactive to heart-specific M7Aα peptide. We found that 3 injections of LPS (5 µg/kg) significantly reduced concentration of IgG reactive to M7Aα (Figure 3E). Thus, LPS-induced TLR4 signaling inhibited T and B effector lymphocyte function in EAM.

**Figure 4:**
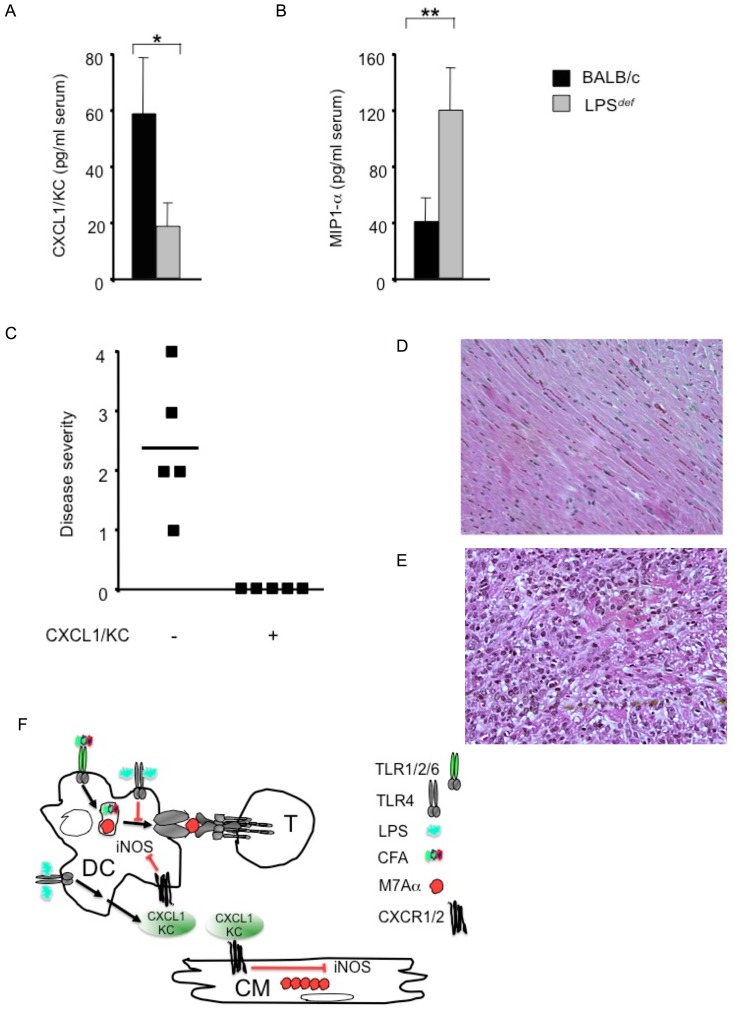
The chemokine CXCL1/KC abrogates experimental autoimmune myocarditis in mice. (A) Severe inflammation in the myocardium was accompanied by persistent presence of the chemokine MIP1α. (B) CXCL1/KC was significantly elevated in the serum of wild type BALB/c mice and significantly reduced in the serum of *LPS* ^def^ mice. Serum chemokine concentrations were determined 21 days after the initial immunization with heart-specific M7Aα peptide in CFA (5 mice per group, Mean value ± SD). (C) Therapeutic administration of mouse recombinant chemokine CXCL1/KC, after experimental challenge with heart-specific M7Aα peptide in CFA, abrogated heart inflammatory infiltrate and heart muscle damage. Squares represent individual mice, lines indicate mean values. (D) Bland histopathologic picture in a representative heart section from a mouse treated with mouse recombinant chemokine CXCL1/KC after challenge with M7Aα peptide in CFA. Original magnifications x10 is shown. (E) Severe myocarditis in a heart section from a control mouse injected with saline after challenge with M7Aα peptide in CFA. Original magnifications x30 is shown Hearts were analyzed 21 days after the initial immunization with heart-specific M7Aα peptide in CFA. Staining was with hematoxylin and eosin (H&E). Mice were immunized with heart-specific M7Aα peptide in CFA and heart histopathology was determined at day 21 after the initial immunization. One result representative of 5 independent experiments is shown. (F) Cartoon outlining the mode of action by systemic LPS and CXCL1/KC in EAM. M7Aα peptide and CFA co-localize within the phagosme where M7Aα peptide is processed for presentation in conjunction with MHC class II molecules. TLR4 is dispensable for auto-antigen processing and presentation, TLR4, in fact, inhibits this process which, in turn, limits the autoantigen dependent expansion of pathogenetic T_effs_. TLR4 promotes the production of CXCL1/KC in EAM; and CXCL1/KC is cardioprotective as exemplified by its prevention of iNOS protein induction. T, effector T lymphocyte; DC, antigen presenting dendritic cell; CM cardiomyocyte.

Interleukin 6 (IL-6)-deficient mice resisted the development of EAM because of their inability to expand pathogenetic T_eff_ cell populations [27]. IL-6 is the pivotal cytokine in mediating differentiation of T_H_17 cells and EAM is a T_H_17 mediated disease [14], [15]. IL-17A promotes the stabilization of chemokine CXCL1/KC mRNA and increases the concentrations of CXCL1/KC protein [28], [29]._ENREF_39 CXCL1/KC is a CXC-type chemokine involved in the activation and trafficking of inflammatory cells and in protecting cells and organs from injury [30]. Thus, we measured the serum CXCL1/KC protein concentration in mice with EAM, and found that *LPS* ^def^ mice had significantly lower serum CXCL1/KC protein concentrations than wild type control mice, 21 days after initial challenge with M7Aα peptide in CFA (Figure 4A). At the same time, we measured the serum concentration of macrophage inflammatory protein-1 α (MIP1α, CCL3) as both MIP1α and CXCL1/KC are MyD88-dependent chemokines [31], [32]. We found that *LPS* ^def^ mice had significantly greater serum MIP1α protein concentrations than wild type control mice, 21 days after the initial challenge with M7Aα peptide in CFA (Figure 4B).

The finding of a correlation between reduced disease severity and increased serum concentrations of CXCL1/KC in mice with EAM led us to test whether increased CXCL1/KC serum concentrations was the cause of decreased disease prevalence and severity in EAM. To test this hypothesis, we administered CXCL1/KC to BALB/c wild type mice after immunization with M7Aα peptide in CFA; i.e., after the immune response has been induced experimentally but before myocarditis was apparent [20]. We found that the regime of 3 intraperitoneal injections of recombinant mouse CXCL1/KC (1 µg/kg), given every other day, starting three days after the boost immunization with M7Aα peptide in CFA, was sufficient to completely prevent cardiac inflammation and cardiomyocyte damage in these immunized mice (Figure 4C,D). Conversely, saline injected control mice developed myocarditis with high prevalence and high severity (Figure 4E).

Macrophages dominate the inflammatory infiltrate in EAM (Figure 1D, Table 1). Macrophages are phenotypically and functionally heterogeneous cells that respond exquisitely to environmental signals [33]. Inducible NO synthase (iNOS) protein expression in macrophages is a hallmark of EAM [34]. Therefore, to test whether systemic administration of CXCL1/KC prevented inflammation within the heart by preventing the recruitment of iNOS expressing macrophages or the induction of iNOS in cardiomyocytes, we determined iNOS protein expression within the myocardium after M7Aα peptide challenge. Therapeutic administration of recombinant mouse CXCL1/KC (1 µg/kg) in mice after immunization with M7Aα peptide in CFA prevented the induction of iNOS protein expression in hearts. In hearts of immunized control mice, however, iNOS expression was strongly upregulated in cardiomyocytes, and in cells of the inflammatory infiltrate, mainly comprised of CD68^+^ macrophages, virtually all of which were iNOS positive (Table 1). Together, these data demonstrate that CXCL1/KC is a potent anti-inflammatory chemokine in EAM (Figure 4E).

## Discussion

The results presented here have uncovered an essential function of TLR4 in the induction of the chemokine CXCL1/KC in an organ-specific autoimmune disease. CXCL1/KC exerts an effective anti-inflammatory effect in a heart-specific autoimmune disease. The “hygiene hypothesis” entails that prior exposure to infectious agents can prevent inflammatory responses, e.g., in atopic disease or inflammatory bowel disease and multiple sclerosis [35], [36]. Interestingly, mild gastroeneritis in patients caused by *E. coli* is associated with reduced risk for cardiovascular disease [37]. Our data provide a mechanism by which LPS, a defined component of *E. coli*, can significantly ameliorate autoimmune inflammatory heart disease. The antagonistic LPS mutant mkLPS mimicked the genetic loss of TLR4 signaling in EAM when given locally in the autoantigen emulsion. These data suggest a mechanism where heart antigen presentation required for T_effs_ expansion is inhibited by TLR4 signaling within the DC. When TLR4 signaling in the endophagosome is deficient, due to genetic inactivation or pharmacological inhibition, heart antigen presentation is enhanced and so is T_eff_ cell expansion. This interpretation of our data is consistent with a model where antigen presentation is controlled by the presence of antigen and TLR ligand simultaneously in the same phagosome [8]. The role of TLR4 in the pathogenesis of CB3 virus-induced myocarditis demonstrated that signaling through multiple receptor pathways is necessary for a cytokine environment that exaggerates viral replication and heart tissue damage [13]. We report here that CXCL1/KC production is TLR4 dependent in the context of autoimmune myocarditis.

Our data suggest two mechanisms by which the TLR4 ligand LPS is curbing inflammation. Systemic low dose LPS reduces, firstly, the expansion of T_effs_ and induces, secondly, the production of the anti-inflammatory chemokine CXCL1/KC. We show that the number of T_eff_ is significantly reduced in LPS treated M7Aα immunized mice. We exclude an increased number of T_regs_ as cause for the amelioration of EAM in LPS-treated mice but find an increased capacity of T_regs_ to suppress of T_eff_ proliferation. An effect of APCs is a possible cause for our finding of reduced T_eff_ number in LPS treated mice can, however, not be excluded. An increased capacity of T_regs_ to suppress T_eff_ proliferation, directly or indirectly via APCs [25], an intrinsic suppression of mitogenic activity of T_effs_, or a T_regs_ independent effect on APCs are possible causes for our finding of reduced T_eff_ number in LPS treated mice. CXCR1 and CXCR2 are the G-protein-coupled receptors for CXCL1/KC [38], and expression of CXCR1 is elevated in cardiomyocytes of the failing left ventricle compared to non-failing hearts [39]. The toll-like microbial pattern-recognition receptors which have a role in the protecting against microbial infection, via their essential function in inducing adaptive immune responses, [40], [41] clearly have an important role in curbing excessive organ specific inflammation [30] [42]. Here we uncover the essential role of TLR4 in inducing the chemokine CXCL1/KC which in turn is sufficient to abrogate inflammatory macrophage homing to the myocardium in the face of auto-aggressive T lymphocyte activation.

Our findings raise a caveat against targeting TLR4 for therapy of organ specific autoimmune disease precisely because of its pleiotropic effects [43]. Indeed, inhibiting TLR4 might be contraindicated in myocarditis due to its essential function in inducing increased CXCL1/KC protein expression in the course of myocarditis.

We set out to clarify the role of the molecular pattern recognition receptor TLR4 in induction, maintenance and resolution of EAM. Our results clearly show that TLR4 is not required for the activation of DCs *in vivo*. Eriksson et el. show that agonists of several TLRs, or CD40L, are sufficient to mature DCs *in vitro* so that these matured DCs pulsed with myosin-derived peptide induce myocarditis in recipient mice [44]. This work by Eriksson et el. confirms that TLRs other than TLR4 are sufficient to activate DCs to induce autoimmune myocarditis. In addition, our new results demonstrates that TLR4 is redundant for that function.

In a report, Nishikubo et al. found that C3H/HeJ mice, with nonfunctional mutated TLR4, were resistant to the myosin-induced autoimmune myocarditis [11]. These findings seem to contradict our own experimental findings. The model, however, used by Nishikubo et al. is very different from the one we used, for the following reasons: Nishikubo et al. immunize mice with 100 µg of porcine cardiac myosin mixed with 1 mg of BCG in IFA into the footpad on day 0 and day 14. We inject 25 µg, not of BCG but of *Mycobacterium tuberculosis* (H37Ra), per mouse and immunization. Furthermore, the antigen, porcine protein, versus recombinant mouse peptide, the time of the immunization boost, day 14 versus day 7 in our model, make it difficult to compare results obtained from the use of these two models since they are so obviously different.

Nishikubo et al. attribute their finding of protection from autoimmune myocarditis to an intrinsic bias towards a T_H_2 phenotype in C3H/HeJ mice [11]. In the model we are using a T_H_2 bias would not be protective: IFN-γ-deficient mice develop fatal autoimmune myocarditis [45] and IFN-γ suppresses EAM [46]. The model we are using for this study represents a model of an organ-specific autoimmune disease associated with a T_H_2 phenotype, in which IL-4 promotes the disease and IFN-γ limits it [47]. At the T cell and APC cell level, however, there is no contradiction between the two models. In both models, TLR4 signaling is not required for the activation of myosin-reactive T cells [11]. Since in both EAM models the activation of heart pathogenic T cells is independent of functional TLR4, it is not surprising that C3H/HeJ develop BCG-porcine myosin induced myocarditis after blocking IL-4 [11].

In human disease, patients with DCM, coding polymorphisms of TLR4 were associated with significantly reduced improvement of left ventricular ejection fraction and left ventricular dilation at the follow-up evaluation when compared with carriers of the wild type gene under the same treatment conditions [48]. It will be of great interest to determine whether these human TLR4 coding polymorphisms increased or decreased TLR4 signaling in patients.

The specific function of TLR4 in regulating the immune response to heart epitopes we describe here could be useful for treating autoimmune inflammatory conditions underlying human heart disease or cardiac ischemia-reperfusion injury. More important, our finding that one defined chemokine, CXCL1/KC, abrogates autoimmune inflammatory heart disease opens the possibility for a new specific treatment for myocarditis and cardiomyopathy.

## Materials and Methods

### Induction of autoimmune inflammatory heart disease in mice

*LPS* ^def^ (C.C3-Tlr4^Lps-d^/J) mice, obtained from The Jackson Laboratory, carry a point mutation in the third exon of the *tlr4* gene, that replaces proline with histidine at position 712 of TLR4 protein rendering these mice profoundly refractory to effects of LPS from gram-negative bacteria [17] by abolishing MyD88- and Trif-dependent signaling [49]. Since *LPS* ^def^ mice carry this mutation on a BALB/c background BALB/cJ mice were used as controls [50]. For all experiments, age matched 6 to 8 wk old female mice, housed under specific pathogen-free conditions at the University of Illinois animal facility, were used. Mice were injected subcutaneously with 100 µg/mouse of the mouse α-myosin heavy chain peptide M7Aα (Ac-SLKLMATLFSTYASADOH) emulsified 1∶1 with Complete Freund's adjuvant (CFA) on days 0 and 7 [6].

### TLR4 ligand treatment

mkLPS, the LPS from a mutant *E. coli* strain (2 µg/immunization/mouse), K12 *msbB*, which lacks the myristoyl fatty acid moiety of the lipid A displays a 1,000-10,000-fold reduction in the ability to activate NF-κB [21] and acts as an antagonist to wild type *E. coli* LPS through direct interaction with TLR4 [22] was administered in the emulsion of M7Aα in CFA at the times of immunization. For low dose LPS treatment, mice were injected i.p. with highly pure LPS from *Escherichia coli* strain O111∶B4, with three single doses 5, 9 and 13 days after the initial immunization.

***in vitro*** **stimulation and suppressor assays** was performed as described [6], [51]. Briefly, spleens were removed 21 days after the first immunization with M7A α, and T cells were enriched by negatively sorting out CD11b-, Gr1-, and B220-expressing cells and, for suppressor assay. T cells were cultured with γ-irradiated syngeneic splenocytes pulsed with M7A α peptide (at 50 µg/ml) and cell number and phonotype were determined as described [6]. For suppressor assay, splenic T cells (1×10^5^), enriched as above, from mice immunized with M7A α in CFA and treated with LPS or saline, were cultured with γ-irradiated syngeneic splenocytes pulsed with M7A α peptide (at 50 µg/ml) as described [6). To determine their suppressor activity and phenotype, cultured T cells (1×10^5^) were added to from T cells (5×10^4^) from naïve mice cultured in U-bottom 96 well plates with anti-CD3ε (clone 145-2C11, hamster IgG), and anti-CD28 (clone 37.51, PharMingen) for 48 h. Proliferation of T cells was determined by incorporation of (^3^H) thymidine for the last 12 h of the culture. Flow cytometric analysis was performed as described using antobodies from Pharmingen and eBioscience (FoxP3) [51].

### CXCL1/KC treatment

BALB/c mice were injected i.p. with recombinant mouse CXCL1/KC (Devatal Inc., Hamilton, NJ) at a dose of 1 µg/kg on days 11, 13, and 15 after the initial immunization with M7α in CFA. For immunoperoxidase staining, cryostat sections were fixed in chloroform (30 minutes), and endogenous peroxidase activity was blocked with NaN3 and H_2_O_2_. After incubation with a rabbit polyclonal antibody reactive to murine iNOS (30 minutes at 4 degrees Celsius; anti-MacNOS antibody; Transduction Laboratories) [34].

### Histopathology and serum IgG measurement

Hearts were removed 21 days after the initial immunization and myocarditis, the presence of inflammatory cells and cardiomyocyte damage, was graded on H & E stained heart sections from 0 to 4. 0 = no inflammatory infiltrates; 1 = small foci of inflammatory cells between myocytes; 2 = larger foci of >100 inflammatory cells; 3 = more than 10% of a cross section involved; and 4 = more than 30% of a cross section involved [20], [44]. Serum was collected 21 days after the initial immunization and IgG antibodies reactive to cardiac-specific epitope M7Aα was determined by ELISA as described [6].

### Statistical analysis

Differences in disease severity, IgG antibody production, chemokine production, and T cell expansion and phenotype were analyzed by Student's t-Test or when more than two cohorts of mice were compared by ANOVA for multiple-sample comparisons (Bonferroni) or by Student's t-Test.

### Ethics statement

The mice were maintained and used in strict accordance with the recommendations in the Guide for the Care and Use of Laboratory Animals of the National Institutes of Health. The protocol was approved by the University of Illinois animal care committee (PHS Animal Welfare Assurance number A3460-01). Mice were sacrificed according to the Guide for the Care and Use of Laboratory Animals of the National Institutes of Health to minimize suffering.
