# Correction: Therapeutic Administration of the Chemokine CXCL1/KC Abrogates Autoimmune Inflammatory Heart Disease

**DOI:** 10.1371/journal.pone.0100608

**Published:** 2014-06-20

**Authors:** The PLOS ONE Staff

The text “_ENREF_2_ENREF_3” incorrectly appears at the beginning of the second sentence of the Introduction and should be deleted. The text “_ENREF_81” also incorrectly appears in the first sentence of the Results section and should be deleted.

Figure 1 is missing sub-figures D, E, and F. Please see the complete, corrected Figure 1 here.

**Figure 1:**
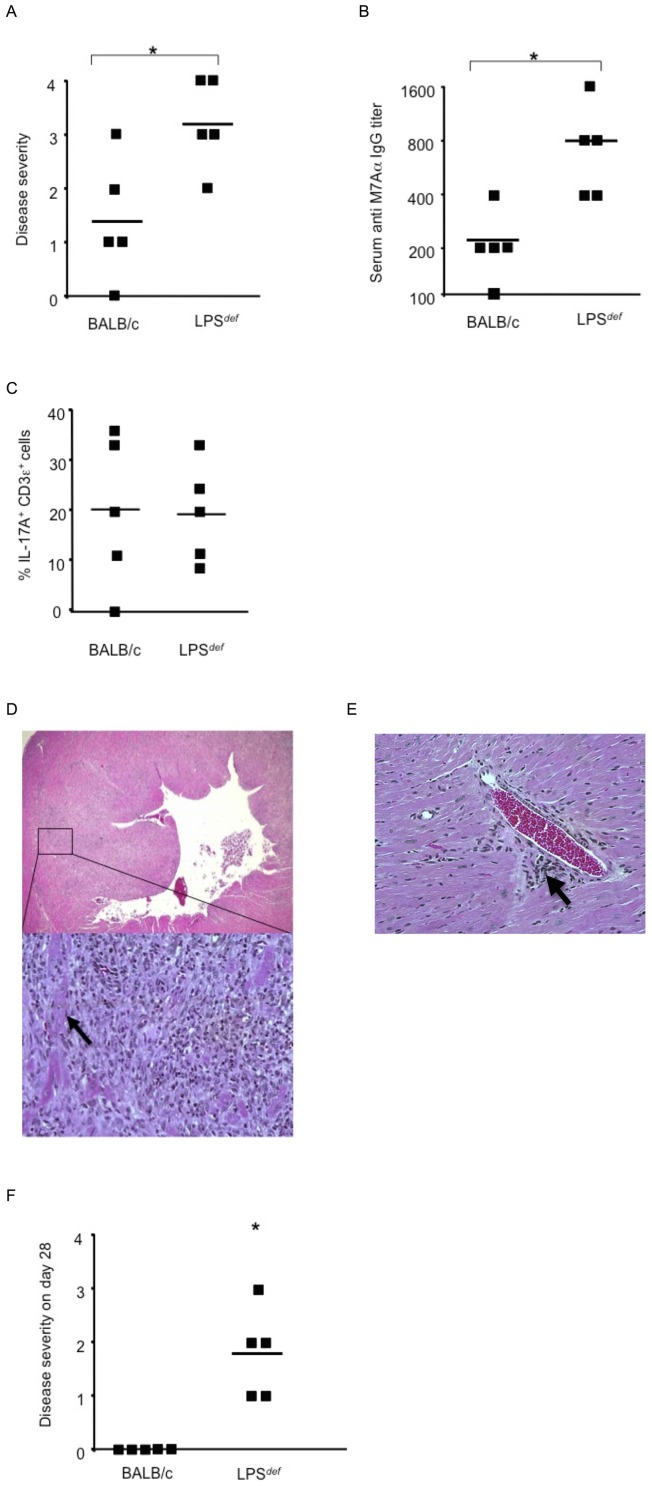
***LPS*** ^def^ mice are highly susceptible to induction of autoimmune inflammatory heart disease. Genetic loss of TLR4 function leads to severe autoimmune myocarditis in mice challenged with heart-specific autoantigen and complete Freund's adjuvant (CFA), a complex mixture of TLR agonists. (A) *LPS* ^def.^ mice lacking functional TLR4 developed significantly more severe autoimmune myocarditis that wild type BALB/c control mice. Histopathological disease severity, as described in Methods, was determined 21 days after the initial immunization with heart-specific M7Aα peptide and CFA. * p<0.05. One representative result out of 5 independent experiments is shown. (B) Serum IgG autoantibodies reactive to heart specific epitope M7Aα were determined 21 days after initial immunization with heart-specific M7Aα peptide and CFA. * p<0.05. One representative result out of 5 independent experiments is shown. (C) Heart inflammatory infiltrate. CD3ε^+^ T cells expressing IL-17A were evaluated 21 days after initial immunization with heart-specific M7Aα peptide and CFA. Squares represent the percentage of IL-17A^+^ cells per CD3ε^+^ T cells as determined by immunohistochemistry in heart-sections from individual mice, lines indicate mean values. * p<0.05. One representative result out of 5 independent experiments is shown. (D) Representative photomicrograph of heart section from a *LPS* ^def^ mouse immunized with autoantigen and CFA. Inflammatory infiltrate consisting mostly of mononuclear cells is present throughout the myocardium often surrounding necrotic cardiomyocytes (arrow). Original magnifications x10 and x200 are shown. Hearts were analyzed 21 days after initial immunization with heart-specific M7Aα peptide in CFA. Staining was with hematoxylin and eosin (H&E). (E) Histopatholgy in a heart section from a BALB/c mouse immunized with heart-specific M7Aα peptide in CFA. Inflammatory infiltrate consisting mostly of mononuclear cells is present as an inflammatory focus (arrow). Original magnifications x100 is shown. (F) *LPS* ^def.^ mice fail to resolve autoimmune myocarditis by day 28 after the initial autoantigen challenge but not wild type BALB/c control mice. Histopathological disease severity, as described in Methods, was determined 28 days after the initial immunization with heart-specific M7Aα peptide and CFA. * p<0.05. Squares represent individual mice, lines indicate mean values. One representative result out of 3 independent experiments is shown. Student's t Test was used for statistical analysis.

## References

[1] Bachmaier K, Toya S, Malik AB (2014) Therapeutic Administration of the Chemokine CXCL1/KC Abrogates Autoimmune Inflammatory Heart Disease. PLoS ONE 9(2): e89647 doi:10.1371/journal.pone.0089647 24586934 10.1371/journal.pone.0089647 PMC3937330

